# Energy-filtered Electron Transport Structures for Low-power Low-noise 2-D Electronics

**DOI:** 10.1038/srep36167

**Published:** 2016-10-31

**Authors:** Xuan Pan, Wanzhi Qiu, Efstratios Skafidas

**Affiliations:** 1 Department of Electrical and Electronic Engineering, University of Melbourne, Parkville, Victoria 3010, Australia; 2 Centre for Neural Engineering (CfNE), University of Melbourne, Parkville, Victoria 3010, Australia

## Abstract

In addition to cryogenic techniques, energy filtering has the potential to achieve high-performance low-noise 2-D electronic systems. Assemblies based on graphene quantum dots (GQDs) have been demonstrated to exhibit interesting transport properties, including resonant tunnelling. In this paper, we investigate GQDs based structures with the goal of producing energy filters for next generation lower-power lower-noise 2-D electronic systems. We evaluate the electron transport properties of the proposed GQD device structures to demonstrate electron energy filtering and the ability to control the position and magnitude of the energy passband by appropriate device dimensioning. We also show that the signal-to-thermal noise ratio performance of the proposed nanoscale device can be modified according to device geometry. The tunability of two-dimensional GQD structures indicates a promising route for the design of electron energy filters to produce low-power and low-noise electronics.

In electron-based transport systems, such as 2-D graphene electronics, the electrons have an energy distribution that is described by the Fermi-Dirac statistic which indicates that the kinetic energy of electrons participating in charge transport varies considerably. Electrons with high kinetic energies have higher momentum and impart higher momentum, upon collision, to the lattice as they traverse the device channel, in the process generating more heat, noise and leading to undesirable effects such as electromigration.

The spatial power dissipation within structures can be calculated using the drift-diffusion approach^1^,^2^,^3^,^4^:


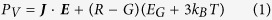


where ***J*** is the current density which counts all carriers that are participating in charge transport, ***E*** is the electric field, (*R*-*G*) is the net non-radiative recombination rate, *E*_*G*_ is the semiconductor band gap, *k*_*B*_ is the Boltzmann constant and *T* is the lattice temperature. The total power dissipated is determined by integrating *P*_*V*_ (power density per unit volume) over the device volume.

The total current density^5^ is given by:


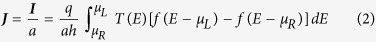


where ***I*** is the current, *a* is the area coefficient, *q* is the electronic charge, *h* is the Planck constant, *T(E)* is the transmission function at an energy level *E*, *μ*_*L,R*_ are the electrochemical potentials of the left and right electrodes, *f* is the Fermi-Dirac distribution of electrons.

The noise density at finite frequency *ω* for the energy band [*E*, *E*+*dE*] is^6^,^7^,^8^:


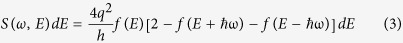


where ℏ is the reduced Planck constant and *f* is the Fermi Dirac distribution. The total current noise is the integral of the noise density across the allowable energy ranges and frequencies. As the power spectral density, *S*(*ω*), is a positive definite function, electron energy filtering is able to help decrease the noise, as is mathematically proved by the following functions^8^:


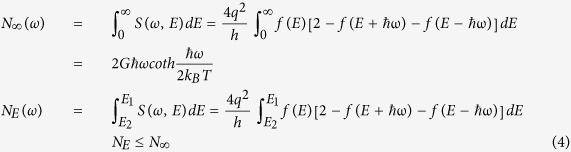


where *G* is the total conductance, *N*_*E*_ is the current noise over a finite energy domain, and *N*_*∞*_ is the generalized Nyquist noise formula which reduces to Johnson-Nyquist thermal noise 4*K*_*B*_*TG* in the limit of 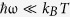 and the quantum zero-point noise 2ℏ*ωG* in the limit of 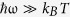.

Reducing energy dissipation and noise in nanoscale electronics are important challenges in the design of 2-D circuits and systems. Considering equations (1, 2, 3, 4), it can be seen that the decrease of noise can be achieved by reducing the energies of electrons that participate in the transport. This is because the total current noise is the integration of the noise density, a positive definite quantity, over all the allowable energy levels. If the integral interval is reduced, the value of the integral, because the noise power spectral density at any energy level is a positive number or zero, will also reduce. The reduction of the noise then leads to the reduction of the signal and consequently the power required to achieve the same signal-to-noise ratio (SNR). Dimensional confinement on devices leads to discrete energy levels^9^,^10^,^11^ which can serve as energy filters as only electrons whose energies match the discrete energy levels are allowed to participate in the tunnelling^12^ and transport, therefore helping to limit the total current noise and power dissipation.

The study of electron transport in QD-based devices has been one of the foci in quantum physics^13^. Because of the quantum confinement, quantum dots have sharper density of state distributions than higher dimensional structures, which has led to them being investigated for use in diode lasers, amplifiers, biological sensors, *etc.*^13^,^14^. Unique electronic properties in quantum dots include: highly nonlinear I-V characteristics, negative differential resistance (NDR) and electrical switching^15^,^16^,^17^. The discrete energy distribution in QDs allows for interesting current-voltage characteristics to be observed where contrary to the conventional Ohmic relationship holding, a decrease in current is noticed with the increase in applied bias when NDR is presented. This property can be considered in building electron energy band-pass filters.

Several methods have been investigated to obtain a pronounced NDR effect, including adsorption of different molecules on quantum dots, varying the shape of quantum dots, use of specific materials as electrodes, *etc.*^18^,^19^,^20^,^21^ Zheng *et al*.^14^ studied the electron transport properties of a C_60_-based electronic device, where two C_60_s were linked by an alkane chain, and highlighted that the NDR can be controlled by the length of the linker; Zhang *et al*.^22^ focused on the transport properties of GQDs sandwiched between two semi-infinite zigzag-edged GNR electrodes, showing that the size of QDs effects the number and position of resonant peaks; Perrin *et al*.^23^ reported upon experiments where a pronounced negative differential resistance was observed in the current-voltage characteristics of a single molecule located in a break junction. In the above-mentioned systems, even though NDR behaviours can be generated, they occur at a relatively high bias (1–2 V), which can limit their application to low power electronics.

In this paper, with a GQD as the basic building block, we construct a fundamental two-terminal energy filter where NDR is obtained at low bias and quantum tunnelling achieves high peak-to-valley current ratio (PVCR). In order to develop a design methodology to control the NDR effect, several versions of the structure are proposed. Doubling GQDs in a symmetrical way can lead to multiple negative resistance regions (and energy passbands), whilst the in-plane parallel GQDs structure causes a displacement of current peak without greatly affecting its magnitude.

## Results and Discussion

**Figure 1:**
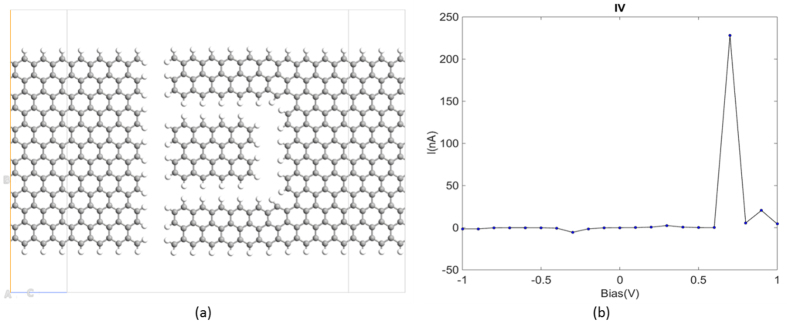
(**a**) The atomic structure of the graphene quantum dot side-gate device; grey and white spheres represent carbon and hydrogen atoms, respectively. (**b**) The calculated I-V curve.

The structure of the elementary GQD device studied here is shown in Fig. 1a. It consists of one zigzag GQD surrounded by two carbon nano-ribbon electrodes. All dangling bonds were passivated with hydrogen atoms. Simulations were performed using ATK from Quantumwise. The atomic structure was fully relaxed using the quick optimization calculation method. After relaxation, the potential of the right electrode was set to be zero and the left electrode was set to be the bias voltage. A pronounced NDR feature is exhibited at a low bias: starting from 0.6 V bias, the current increases initially, reaching a maximum of about 230 nA at 0.7 V, thereafter it sharply decreases (see Fig. 1b). The PVCR is defined as follows:


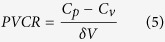


where Cp and Cv are peak and valley currents respectively, and δV is the corresponding voltage difference. The PVCR in our proposed device is approximately 2300 nA/V.

**Figure 2:**
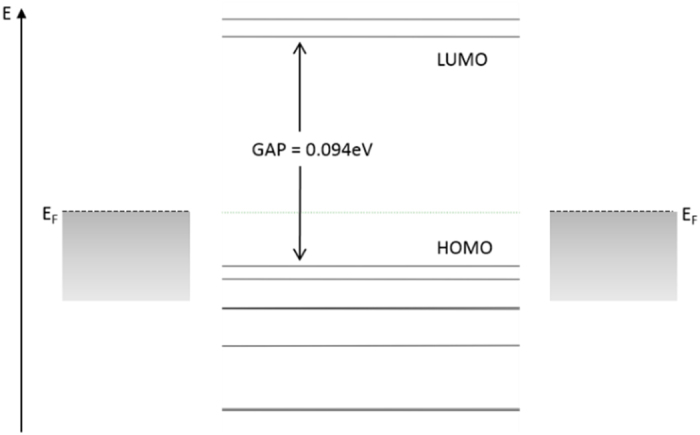
The schematic band diagram showing the energy levels in the bulk GQD side-gate device and the Fermi level (E_F_) in the contacts. The highest occupied molecular orbital (HOMO) was separated by a gap of 0.094 eV from the lowest unoccupied molecular orbital (LUMO).

To understand the operation of the GQD side-gate device, the equilibrium energy level diagram, with the assumption that no voltage was applied between the source and the drain, was illustrated in Fig. 2. A central factor in determining the current flow through the GQD is the location of the Fermi energy of the contacts relative to the energy levels of the GQD. Current flow requires the source to inject carriers into the GQD energy levels. The current is high if the Fermi level of the source aligns with one of the unoccupied energy levels^24^. However, this is not the case under equilibrium, where the Fermi level always lies close to the charge neutrality level near the center of the gap, leading to minimal current at contact/GQD interfaces. The onset of current flow occurs when electrons gain sufficient energy to tunnel from the chemical potential of the source to the LUMO. Under bias, the energy of the LUMO and the Fermi level of the source can be shifted by electrostatic and charging-induced changes in potential. So as the drain-source bias increases, when the chemical potential of the source becomes close enough to the LUMO energy, charging on the GQD is non-negligible, and the current increases with the GQD charge. Finally, a maximum charge density is reached when the chemical potential is aligned with the LUMO, which is reflected as the current peak at 0.7 V in Fig. 1b. Further increases in applied bias lead to a decrease rather than an increase of the charging energy or the current flow, owing to the discrete band structure of the GQD and the misalignment between energy levels.

**Figure 3:**
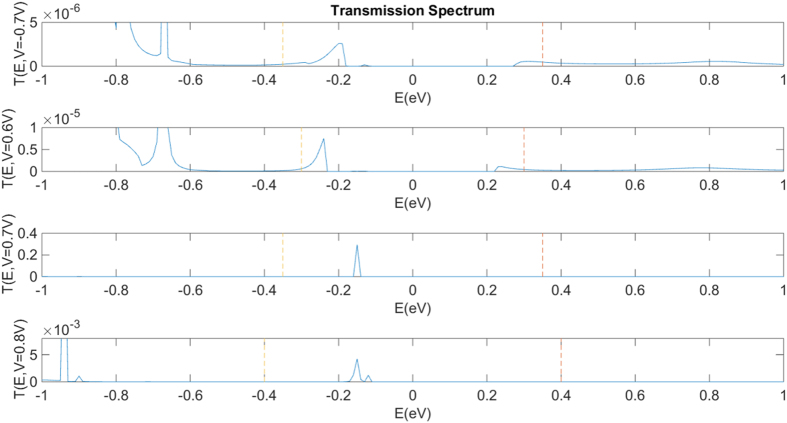
The evolution of the transmission spectrum as a function of bias voltage in the GQD side-gate device. The two vertical lines indicate the bias window.

The transmission functions T(E) for four biases were calculated and are plotted in Fig. 3. According to the Landauer formula, T(E) is the rate at which electrons with energy E are transmitted from the source to the drain by propagating through the device^25^,^26^. For a given bias voltage, the current can be calculated by integrating T(E) across the energy range of the bias window. In Fig. 3, with the bias voltage fixed at 0.7 V, a sharp peak is located inside the bias window with an energy value of −0.15 eV, relative to the Fermi level, and the T(E) value being 0.3. This value of the transmission function is larger than the corresponding ones under the other three simulated bias voltages (−0.7 V, 0.6 V and 0.8 V) by at least two orders of magnitude.

The physical explanation for the features in T(E) can be derived from the density of states (DOS). The DOS shows the number of energy eigenstates in a material per unit energy, and is proportional to the quantum capacitance^5^. At certain energy level, the larger the DOS is, the larger the quantum capacitance will be, and the more charge can be transferred without shifting the level. From Fig. 2 we can see that the GQD has a non-uniform density of states. Darker and thicker energy lines reflect increased DOS. Therefore, the transmission function varies depending on energy position, leading to peaks and valleys in the transmission spectrum.

Based on the structure’s transmission spectrum at 0.7 V and at 300 K, the Nyquist thermal noise power with the GQD filter is −117 dB, whilst without the filter is equal to −51 dB.

**Figure 4:**
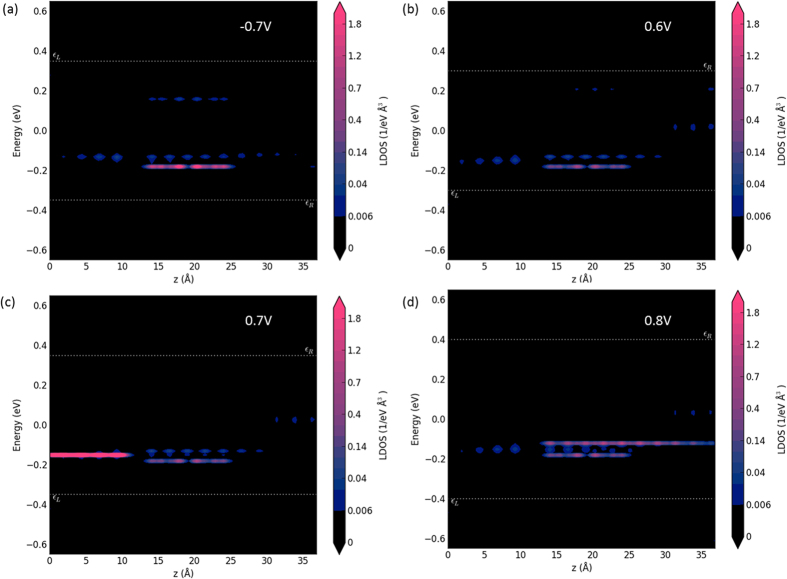
The position-dependent LDOS as a function of bias voltage in the GQD side-gate device. (**a–d**) are position-dependent LDOS plots under −0.7 V, 0.6 V, 0.7 V and 0.8 V bias voltages at −0.2 eV, −0.25 eV, −0.15 eV, and −0.15 eV respectively. The variable z indicates position along the transport direction.

Position-dependent local density of states (LDOS), which are plotted in Fig. 4 for various biases, offers another explanation for the occurrence of the NDR at 0.7 V bias. The LDOS can be determined by solving time-independent Schrodinger equation^27^:


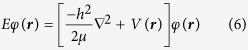


where *φ*(***r***) is the wave function of the quantum system, *μ* is the reduced mass, ∇^2^ is the Laplacian, *V*(***r***) is the electrostatic potential energy, and *E* is the energy which directly corresponds to LDOS. Both *φ*(***r***) and *V*(***r***) are position-dependent. *V*(***r***) can be written as:


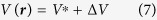


where V* is a constant that denotes the structure’s intrinsic electrostatic potential and Δ*V* is the variation of electrostatic potential due to the applied bias voltage. In Fig. 4 the high number of LDOS manifests in the left electrode only when a bias of 0.7 V is applied. It indicates that under this condition, electrons have a much greater probability of tunnelling from source, GQD and then drain, which contributes to the transmission peak in the transmission spectrum plots (Fig. 3). The integration window and the distribution of the transmission function give rise to the NDR peak at 0.7 V in the I-V curve.

**Figure 5:**
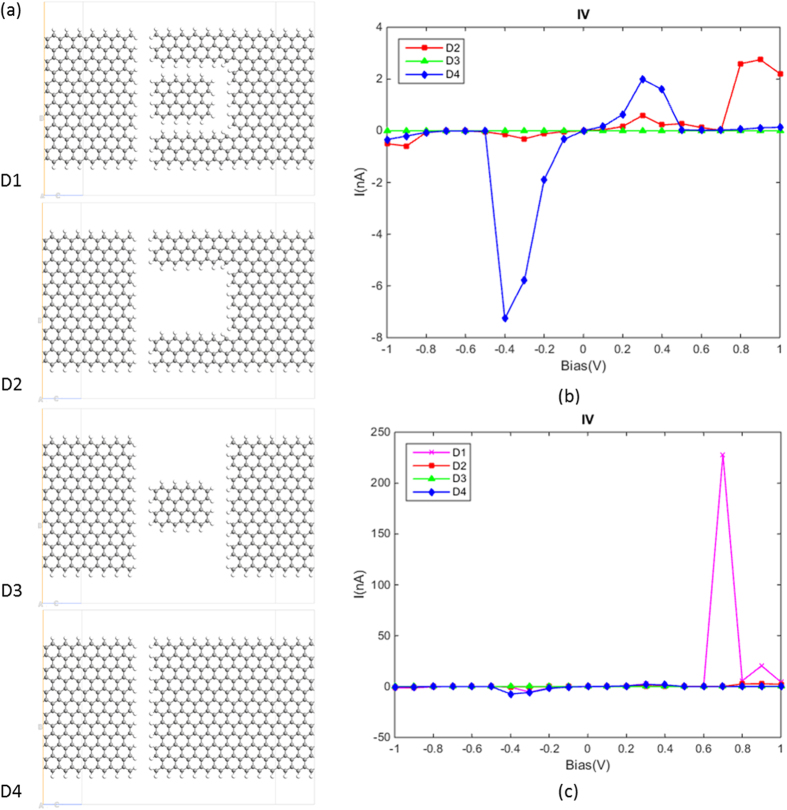
(**a**) Atomic structures of four different devices. (**b**) The calculated I-V curves for D2, D3, D4. (**c**) The calculated I-V curves for all four devices.

In Fig. 5a we show four comparable simulated structures, in which D1 is the key structure proposed here for energy filtering, whilst D2, D3, and D4 are all variants of the structure D1. The GQD and side gates were removed in D2 and D3 respectively, whilst D4 contains only one uniform gap with a width of 0.3 nm. From the plots (see Fig. 5b,c), we can establish that the sharp peak occurs in structure D1 (The inclusion of side gates and quantum dot in combination with the nanoribbon control NDR effect).

Based on equation (4), the signal-to-thermal noise ratio for structures D1–D4 were calculated at room temperature (300 K) (see Table 1). D1 exhibits the best signal-to-thermal noise ratio performance because of its lowest thermal noise power and highest signal power, which is, in essence, attributed to the energy filtering and tunneling effect of the quantum dot structure. By adjusting the device configuration, the noise and signal power can be tuned separately. For example, D1 and D4 have approximately the same thermal noise power, whilst the signal power for D1 is higher by two orders of magnitude. D2 and D4, conversely, have more obvious difference lie in the thermal noise power.

**Figure 6:**
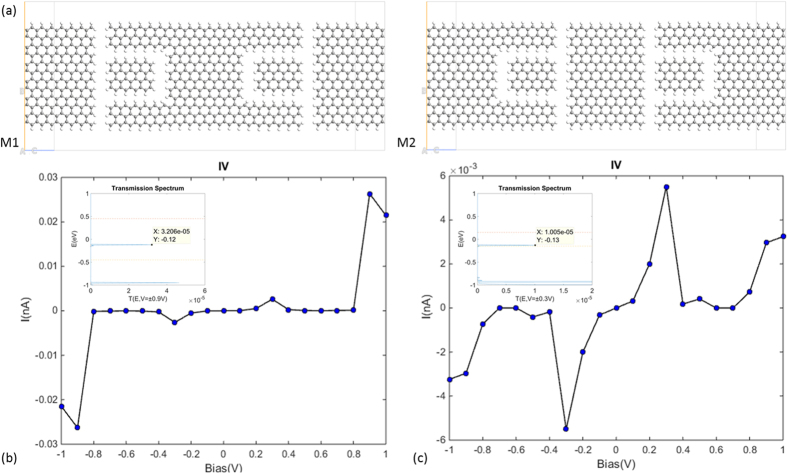
(**a**) Atomic structures of two geometrically symmetrical devices. (**b**) The calculated I-V curve for M1. Inset, the transmission spectrum under ±0.9 V bias voltages. The peak of the energy passband locates at −0.12 eV with the T(E) value being 3.2 × 10^−5^. (**c**) The calculated I-V curve for M2. Inset, the transmission spectrum under ±0.3 V bias voltages. The peak of the energy passband locates at −0.13 eV with the T(E) value being 1.0 × 10^−5^. The two horizontal lines indicate the bias window.

Since the NDR is affected by electrostatic distribution, its magnitude and position can be varied by changing the distribution of electrical field. As examples, we considered two kinds of symmetrical extensions on the central region of the fundamental device (see Fig. 6a). The role of the added GQD is to shift the Fermi level and build symmetry between the forward and reverse currents. After extension, double and multi-NDR effects were achieved. In contrast to the single GQD-based device, the I-V curves for the new structures are symmetrical for positive and negative bias. In M1 the positions of the current peaks shifted to ±0.9 V whilst in M2 the NDR effects appeared most prominently at ±0.3 V and ±0.9 V (see Figs 5c and 6b). The transmission spectrum plots (insets of Fig. 6b,c) reveal that the magnitudes of the energy passbands were tuned largely with little impact on their positions.

**Figure 7:**
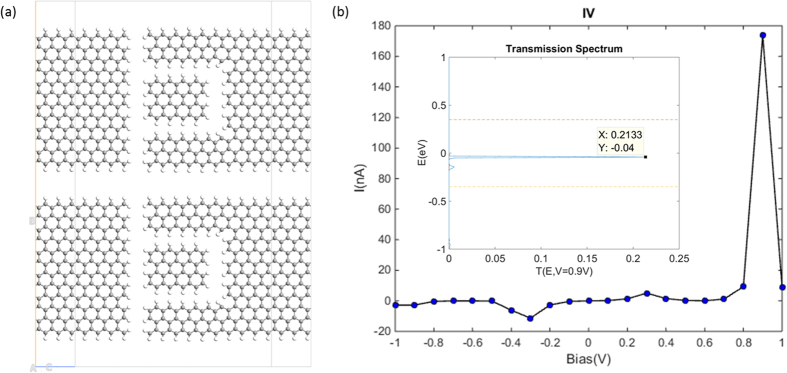
(**a**) The atomic structure of the double parallel-connected device. (**b**) The calculated I-V curve. Inset, the transmission spectrum under 0.9 V bias. The two horizontal lines indicate the bias window.

Furthermore, an in-plane parallel connection of multiple devices turned out to be able to tune the positions of the current peak as well as the energy passband without significantly influencing their magnitudes. In Fig. 7b, the NDR started from 0.9 V bias with the maximum current reaching 170 nA, and the energy passband occurred in the domain of [−0.05 eV, −0.03 eV] with the peak value of 0.2.

## Conclusion

Reduction in power consumption, reliability and noise are important considerations in 2-D electronics. In this paper we show that electron filtering and NDR can be achieved by nanostructuring of conductors and this can be exploited to reduce noise in electronic devices. We proposed several configurations of two-terminal devices with GQD(s) as the basic building block and investigated their transport properties. We found that the electron conduction is mediated by the electrostatic potential distribution which attributes to the joint effect of the device structure and the external applied electric field. As the distribution varies, energy filtering and the NDR effects appear. It is important to note that the proposed symmetrical multi-NDR effect can be realized by mirroring GQD based on different axes or in other ways that affect the relative positions of Fermi levels of electrodes and GQDs. The magnitude and position of energy passbands and the signal-to-thermal noise power ratio can also be tuned with the adjustment of the device geometry. The possibility of controlling both the energy filtering and the NDR effect provides design flexibility when devising GQD based electronic devices and can be used to adapt structures to meet requirements on low power consumption and low current noise.

## Methods

The model structures studied here are constructed as follows: in a two-terminal side-gate device, the metallic side gates in the device are graphene nanoribbons (GNRs) with widths of 6 carbon atoms, while the semiconducting channel is a zigzag GQD with a width of 8 carbon atoms. The whole system is divided into three parts: the semi-infinite left electrode (L), the semi-infinite right electrode (R), and the central scattering region (C). The device geometry has been optimized and the coordinates have been relaxed using the Brenner potential^28^ with the maximum interatomic force no more than 0.05 eV/Å.

The calculations are carried out using the Extended Huckel Method as implemented in ATK-SE package^29^. The mesh points are defined as uniformly spaced k points of 1 × 1 × 50 for all devices, with 50 sample points along the transport direction of the two-terminal structure. The conductance and nonlinear I-V characteristics are studied by the non-equilibrium Green’s Function (NEGF) formalism^30^,^31^ based on optimally localized orbitals^32^,^33^. The transmission function is obtained from the Landauer formula^34^:


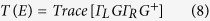


where *G* and *G*^+^ are the retarded and advanced Green’s functions of the central scattering region respectively, and *Γ*_*L,R*_ describes the coupling of left/right electrodes with the central part. The coherent current through the system is given by the integration of the transmission function over the bias window:


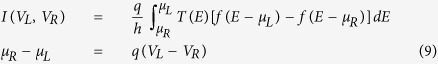


where *V*_*L,R*_ are the left and right electrode potentials respectively, *q* is the electronic charge, *h* is the Planck constant, *μ*_*L,R*_ are the electrochemical potentials of the two electrodes, *f* is the Fermi-Dirac distribution of electrons.

## Additional Information

**How to cite this article**: Pan, X. *et al*. Energy-filtered Electron Transport Structures for Low-power Low-noise 2-D Electronics. *Sci. Rep.* **6**, 36167; doi: 10.1038/srep36167 (2016).

**Publisher’s note:** Springer Nature remains neutral with regard to jurisdictional claims in published maps and institutional affiliations.

## Figures and Tables

**Table 1:** The signal-to-thermal noise ratio for structures D1, D2, D3 and D4.

Structures	Thermal noise power (w)	Signal power (w)	Signal-to-thermal noise ratio (dB)
D1	2 × 10^−12^	1.6 × 10^−7^	49.1
D2	1.3 × 10^−11^	2.43 × 10^−9^	22.7
D3	4.6 × 10^−7^	1.2 × 10^−16^	−95.9
D4	3.3 × 10^−12^	2.8 × 10^−9^	29.2
